# Supplementary material for: Psychosocial well-being and mental health of low- and middle-income countries’ internally displaced persons and refugees during COVID-19: a systematic literature review
Source: Glob Ment Health (Camb). 2024 Dec 10;11:e122. doi: 10.1017/gmh.2024.110 (PMC11704375; doi:10.1017/gmh.2024.110)
Supplement: Amodu et al. supplementary material 1 — Amodu et al. supplementary material [file S2054425124001109sup001.pdf]

Extraction Table

**Author(s) and Publication Year**	**Purpose**	**Design**	**Setting and Population**	**Findings**
Akhtar et al., 2021	To examine the impact of the COVID-19 pandemic on the mental health of Syrian refugees living in Azraq Camp in Jordan.	Quantitative: A longitudinal cohort study used data from a randomized controlled trial (RCT) before and during the pandemic to compare mental health outcomes.	**Setting:** Azraq Refugee Camp, Jordan<br>**Population:** Syrian refugees	1. The results show refugees were generally concerned about economic difficulties (82.4%), shortages of basic supplies (71.3%), and fears of infection for themselves or others.<br>2. Compared to assessments before the pandemic, post-traumatic stress disorder (PTSD) symptoms in refugees were reduced during the pandemic.<br>3. Anxiety or depression scores did not show significant changes based on the timing of the pandemic.<br>4. Pandemic-related anxiety levels were high, and this was an important predictor of pandemic-related concerns.
Alnaji et al., 2024	To investigate the perceived stressors that affect the mental well-being of postpartum Syrian mothers living in Lebanon's Bekaa Valley during the first year of the COVID-19 pandemic.	A qualitative study with interviews of mothers.	**Setting:** Lebanon<br>**Population:** Syrian refugee mothers.	The study identifies four main stressors impacting the mental health of postpartum Syrian mothers.<br>1. Poor living conditions in informal settlements caused significant distress.<br>2. The economic crisis in Lebanon worsened their financial struggles, making it difficult to afford basic necessities.<br>3. Barriers to accessing healthcare, including high costs and legal issues, negatively affected their well-being.<br>4. The pandemic and subsequent lockdowns led to increased social isolation and anxiety.

Extraction Table

Alpay et al., 2021	To investigate how traumatic stress related to the COVID-19 pandemic affects executive functions in Syrian refugees residing in Turkey	Quantitative study. A longitudinal cohort study among 417 Syrian refugees using surveys.	**Setting:** Turkey<br>**Population:** Syrian Refugees	1. The results showed that COVID-19 traumatic stress affected deficits in working memory and inhibitory control directly and indirectly through PTSD and depression.<br>2. The study also found that people infected with COVID-19 had significantly higher deficits in working memory and inhibitory control than those who were not infected.<br>3. These findings highlight the severe impact of COVID-19 traumatic stress on cognitive function and its clinical significance.
Anwar et al., 2023a	To assess the stigma associated with COVID-19 among older adults in Rohingya refugee camps in Bangladesh and its related factors.	A Cross-sectional study were collected from 864 participants at a single point in time through face-to-face interviews.	**Setting:** Rohingya refugee camps in Cox's Bazar, Bangladesh.<br>**Population:** Older refugee adults.	1. The study found that high prevalence of COVID-19-related stigma among older adults in the Rohingya refugee camps.<br>2. Factors associated with higher stigmatization included having formal education, relying on family for support, living farther from health centers, decreased household income during the pandemic, having friends or relatives infected with COVID-19, and reduced communication during the pandemic.<br>3. The study suggests increasing COVID-19 awareness through health education and media outreach to reduce stigmatization among the elderly.
Anwar et al., 2023b	To examine the prevalence of COVID-19-related	Mixed methods: A cross-sectional study conducted	**Setting:** Rohingya refugee camps in Cox's Bazar, Bangladesh.	1. The findings revealed that 68% of participants experienced COVID-19-related anxiety, and 93% felt stress.

Extraction Table

	anxiety and perceived stress among older Rohingya adults, and identify the associated factors.	among 864 Rohingya refugees aged 60 years and above.	**Population:** Older refugees	2. Higher anxiety was associated with challenges in accessing food and healthcare, lack of physical activity, concerns about COVID-19, affected friends or family, and greater stress from losing partners or experiencing COVID-related fears.<br>3. The results underscore the urgent need for psychosocial support for elderly Rohingya refugees.
Atak et al., 2023a	To investigate the prevalence of depression and anxiety among Syrian immigrant pregnant women in Turkey and to identify factors associated with these conditions.	Quantitative: A cross-sectional study.	**Setting:** Sultanbeyli Empowered Migrant Health Center in Istanbul, Turkey.<br>**Population:** Syrian immigrant pregnant women	1. Most participants had minimal anxiety and depression, likely due to effective social support and health services.<br>2. Smoking and first pregnancies were linked to anxiety, while smoking and deliveries beyond 42 weeks were linked to depression.<br>3. Addressing vaccine hesitancy and information gaps, as well as tackling loneliness and language barriers, can enhance psychosocial support for pregnant immigrant women.
Atak et al., 2023b	To explore the prevalence and associated factors of postpartum anxiety and depression among Syrian migrant women living in Turkey.	A Cross-Sectional Study among Syrian migrant women in Turkey.	**Setting:** Strengthened Migrant Health Center in Istanbul, Turkey.<br>**Population:** Surian migrant women	1. The study found generally low rates of postpartum anxiety and depression among Syrian migrant women in Turkey.<br>2. Key factors affecting depression were lower income and COVID-19 vaccination status, while anxiety was linked to lower education levels. More cohabitants were associated with lower depression scores. Despite regular health service use and

Extraction Table

				good health reports, COVID-19 vaccination rates were low.<br>3. The results highlight the need for economic support, educational interventions, and better healthcare access could help address mental health issues.
Badrfam and Zandifar, 2021	To highlight the critical mental health issues faced by Afghan immigrants in Iran during the COVID-19 pandemic.	Discussion paper. Letter to the editor.	**Setting:** Iran<br>**Population:** Afghan immigrants and refugee populations.	1. The article highlights the severe mental health challenges faced by Afghan immigrants in Iran, mainly due to socioeconomic factors and stressful working conditions.<br>2. Iran's economic issues have raised healthcare costs, significantly impacting Afghan immigrants. The COVID-19 pandemic has worsened these problems, increasing stress, uncertainty, and social restrictions, further exacerbating their mental health challenges.
Baluku et al., 2023a	To investigate the relationship between psychological rigidity and post-traumatic stress disorder symptoms and compliance with COVID-19 control measures among refugees in Uganda.	A Cross-Sectional Study surveyed 352 refugees.	**Setting:** Uganda.<br>**Population:** Refugees	1. The study found that higher psychological inflexibility is associated with more severe PTSD symptoms among refugees, and these severe PTSD symptoms negatively impact adherence to COVID-19 control measures.<br>2. Higher psychological inflexibility directly correlates with poorer adherence to these health measures.

Extraction Table

Baluku et al., 2023b	To examine whether psychological rigidity has had a negative impact on the quality of life of refugees in Uganda during the COVID-19 pandemic.	A Cross-Sectional Study surveyed 402 refugees.	**Setting:** The Kampala metropolitan area and the Bidibidi refugee settlement in the West Nile region, Uganda. **Population:** refugees	1. The study confirms that psychological inflexibility negatively impacts quality of life (QoL) among refugees by influencing coping strategies, perceived threat, adherence to control measures, and mental health. 2. Addressing psychological inflexibility through targeted interventions could improve coping and adherence, ultimately enhancing QoL during crises.
Baluku, 2023	To investigate the role of psychological capital in enhancing the quality of life among refugees in Uganda during the COVID-19 pandemic.	Cross-sectional study.	**Setting:** Bidibidi refugee settlement, remote Yumbe district in Northwestern Uganda. **Population**: refugee	1. Psychological capital directly improves the quality of life of refugees; the higher the psychological capital, the better the quality of life. 2. The study supports a sequential mediation model where higher psychological capital enhances positive coping strategies and adherence to COVID-19 preventive measures, which positively impact psychological health, reducing stress and improving overall well-being and quality of life.
Bellizzi et al., 2021	To assess how the COVID-19 pandemic has exacerbated mental health issues among vulnerable populations in Jordan,	Discussion/Opinion paper.	**Setting:** Jordan **Population:** Syrian refugees, displaced populations, and vulnerable Jordanians	1. The article highlights that the COVID-19 pandemic has significantly worsened mental health conditions in Jordan, especially for refugees and migrants, due to increased distress and mental health service disruptions. 2. Refugees and low-income migrants face severe mental health challenges due to job

Extraction Table

	particularly focusing on refugees and displaced individuals.			losses and reduced income, with children's well-being also suffering.<br>3. Recommendations include strengthening mental health services, increasing funding, and integrating these services into broader health frameworks.
Ben Abid et al., 2023	To determine the prevalence of psychological distress among undocumented sub-Saharan African migrants in Tunisia, evaluate potential risk factors, including sociodemographic characteristics, as well as the knowledge and attitudes towards COVID-19 during the early onset of COVID-19 pandemic.	Quantitative cross-sectional design: questionnaires of Pre-COVID-19 pandemic background characteristics, COVID-19 pandemic stressors, and mental health scores. The final sample included 133 respondents with a final response rate of 92%	**Setting:** Tunisia<br>**Population:** Undocumented sub-saharan African migrants	1. The prevalence of psychological distress among sub-Saharan African immigrants in Tunisia is 35%. Immigration processes and other related stressors exacerbate mental health problems. Respondents generally lack relevant knowledge, especially about COVID-19 transmission methods and preventive measures.<br>2. Multiple regression analysis showed that there is a significant association between the level of knowledge about COVID-19 and psychological distress. Respondents with lower levels of knowledge reported higher levels of psychological distress.<br>4. The report shows that immigrants who receive social support have better mental health status, and the dissemination of COVID-19-related knowledge and mental health support to immigrants can alleviate the negative impact of the pandemic on this group.
Bernardi et al., 2021	To investigate how COVID-19-related life changes impact the mental health of Syrian refugees	Longitudinal cohort study involves a two-wave Syrian refugee mental health panel at	**Setting:** Istanbul, Turkey.<br>**Population:** Syrian refugees	1. The study found that COVID-19 stressors, including changes in social relationships, pandemic-related stress, and feelings of hopelessness, were linked to increased depression, anxiety, and stress among Syrian refugees.

Extraction Table

	in Istanbul and to explore the bidirectional effects between pandemic-related stressors and mental health symptoms.	two different points.		2. The research highlighted that pandemic-related stress and poor social connections worsened mental health, and anxiety symptoms would increase pandemic-related stress, pandemic-related stressors affected both men and women similarly.<br>3. The findings underscore the need for targeted mental health support for refugees, emphasizing the importance of improving access to mental health services and addressing socio-economic challenges exacerbated by the pandemic.
Boiko et al., 2024	To investigate and highlight the mental health and sleep disturbances experienced by Ukrainian refugees due to the ongoing conflict between Russia and Ukraine.	A cross-sectional study with 174 individuals participated.	**Setting:** Various countries.<br>**Population:** Ukrainian refugees in various countries worldwide who fled Ukraine due to the full-scale Russian invasion, aged over 18, and could answer the questionnaire in Ukrainian.	1. The study found that Ukrainian refugees had significantly higher levels of obsessive-compulsive symptoms, depression, hostility, and overall psychological distress compared to those who stayed in Ukraine.<br>2. Key factors for mental health issues included job loss, dissatisfaction with living conditions, and social support. Refugees faced greater risks of severe depression, anxiety, and stress, though insomnia rates were similar between the groups.<br>3. The study highlights the urgent need for targeted social support and mental health programs to aid the adaptation and integration of Ukrainian refugees into host societies.

Extraction Table

Budak et al., 2021	To examine the impact of COVID-19 risk perception on the psychological well-being of Syrian refugees and how this relationship varies across different socio-demographic characteristics.	A cross-sectional study via Google Form questionnaire.	**Setting:** Elbeyli Temporary Accommodation Center (TAC) in Kilis and within the province of Kilis in Turkey. **Population:** Syrian refugees.	1. The study found that COVID-19 risk perception significantly affects the psychological well-being of Syrian refugees, explaining 21% of the total variance in well-being scores. 2. Significant differences were observed in risk perception and psychological well-being based on marital status, educational level, and monthly income. 3. Those perceiving higher COVID-19 risks experienced lower psychological well-being, underscoring the importance of providing targeted support to enhance the mental health of Syrian refugees during the pandemic.
Bukuluki et al, 2020	To identify the socioeconomic and psychosocial impact of the pandemic on urban refugees.	Qualitative: discussion paper	**Setting:** Kampala City and other towns in Uganda **Population:** Refugees	Financial pressure, exclusion from food aid because of a lack of identification, restriction of social gatherings, and closure of community-based support are challenges to emotional and psychosocial support.
Caliyurt, 2021	To explain how the United Nations High Commissioner for Refugees (UNHCR) adjusted its Mental Health and Psychosocial Support (MHPSS)	Discussion paper.	**Setting:** Multiple countries. Refugee camps and communities across multiple countries, focusing primarily on the actions of the UNHCR during the	1. UNHCR and its partners continued to provide mental health and psychosocial support to refugees through community mobilization, remote support, psychological first aid training, and enhanced hotlines. 2. These measures included remote psychological first aid via phone and tech, mobilizing community health volunteers, setting up mental health support hotlines,

Extraction Table

	activities during the COVID-19 pandemic.		COVID-19 pandemic.<br>**Population:**<br>Refugees	and disseminating positive coping mechanisms through radio and drama.<br>3. These adaptive practices effectively addressed pandemic challenges and ensured mental health care for refugees under extreme conditions.
Cuijpers et al., 2022	To investigate the effects of a WHO-guided digital health intervention for depression in Syrian refugees in Lebanon	Quantitative paper: single-blind and randomized clinical trial.	**Setting:**<br>Lebanon<br>**Population:**<br>Syrian refugees.	1. WHO-guided digital health intervention by the name “step-by-step” for depression effectively reduced depression symptoms among Syrian refugees in Lebanon.<br>2. The study also found that the participants received the digital intervention well, and adherence to the intervention was high.<br>3. The study suggested that digital health intervention could be a feasible and effective way to deliver mental health services to refugees in humanitarian settings.
Desie et al., 2021	To explore and analyze the coping strategies used by Ethiopian migrant returnees who were placed in mandatory quarantine in Addis Ababa during the COVID-19 pandemic.	A center-based cross-sectional study was conducted with 405 migrant returnees.	**Setting:**<br><br>Addis Ababa, Ethiopia.<br>**Population:**<br>Ethiopian migrant returnees.	1. Results showed that Ethiopian returnees and refugees commonly used religious and emotion-focused strategies in stressful situations, with high rates of such strategies associated with a lack of family support and lack of exposure to COVID-19-infected individuals.<br>2. Planning and problem-solving were second, while dysfunctional coping strategies such as medication were least used.<br>3. The findings highlight the need for a comprehensive support system to address

Extraction Table

				economic and mental health needs and underscore the importance of strengthening adaptive coping strategies among returnees.
Ekoh et al., 2021	To explore the impact of COVID-19 on older people in displacement.	Qualitative paper: Semi-structured interviews from 12 older internally displaced people	**Setting:** Durumi camp, Abuja. Nigeria **Population:** Older IDPs	Economic and psychosocial fallout of the COVID-19 pandemic increased older persons in displacement poverty, psychological stress and placed them at risk of ageism, and social isolation and subsequently leading to secondary displacement, thereby losing all progress, development and resilience built after initial displacement.
El-Khatib et al., 2020	To evaluates the mental health impact of COVID-19 and lockdown measures in Jordan, focusing on refugees in the Zaatari camp, and highlights the need for further support.	Discussion paper.	**Setting:** Zaatari Refugee Camp, Jordan. **Population:** refugees	1. Both the general population and refugees in Jordan face mental health challenges due to pandemic uncertainties and limited healthcare access. 2. Support measures include psychological assistance, online stress management, and adapted health services. In Zaatari camp, mental health support is provided via phone and online resources. Though there was a slight increase in mental health cases during the lockdown, it was not statistically significant. 3. Jordan's response underscores the need for additional resources to tackle the pandemic's economic and mental health impacts.
Erol and Batgi., 2023	To investigate refugee adolescents' quality of life and	A cross-sectional study included 301 Syrian	**Setting:** Istanbul, Turkey. **Population:**	1. The study found that depressive symptoms and personality traits in Syrian refugee adolescents during the COVID-19 pandemic are both significantly

Extraction Table

	its association with depression and quality of life.	refugee adolescents.	Syrian refugee adolescents.	associated with lower quality of life, with depression mediating the relationship between personality dysfunction and quality of life. This indicates that poor personality traits lead to depression, which in turn reduces quality of life.<br>2. The study emphasizes the importance of addressing depressive symptoms and personality issues to improve the quality of life for this population.
Espinel, 2020	To outlines the multiple factors contributing to the mental health struggles of Venezuelan migrants, including the political and economic crises in Venezuela, the hardships of migration, and the impact of the COVID-19 pandemic.	Discussion paper.	**Setting:** Columbia<br>**Population:** Venezuelan migrants	1. The crisis in Venezuela, marked by economic collapse and a failing healthcare system, has led to mass migration. Migrants face severe losses and dangers on their journey and struggle with unemployment and service access upon arrival, worsening food insecurity and economic hardship.<br>2. The COVID-19 pandemic has intensified the psychological stress of Venezuelan migrants in Colombia.<br>3. The article highlights the urgent need for effective mental health and psychosocial support for these migrants.
Fouad et al., 2020	To highlights how the multiple crises facing Lebanon: political stagnation, economic collapse and the ongoing	Discussion paper.	**Setting:** Lebanon.<br>**Population:** Refugees and vulnerable groups.	1. The article highlights the serious mental health challenges facing Lebanon amidst political stagnation, economic collapse, and the ongoing refugee crisis exacerbated by the COVID-19 pandemic, especially for Syrian refugees facing

Extraction Table

	refugee crisis, have placed enormous pressure on Lebanon's mental health infrastructure, especially for refugees and vulnerable groups.			economic hardship and poor living conditions.<br>2. Despite current barriers such as cultural exclusion, infrastructure limitations, and funding constraints, this article argues that tele-mental health (TMH) is a viable solution to bridge the huge mental health service gap in Lebanon.
Green et al., 2023	To evaluate the feasibility of a cluster randomized trial for a community-based psychosocial intervention "Entre Nosotras" designed for refugee, migrant, and host community women in Ecuador and Panamá.	Mixed-methods evaluation of a group psychosocial intervention.	**Setting:** Guayaquil and Tulcán, Ecuador. Panamá City and Panamá West, Panamá.<br>**Population:** Refugee, migrant, and host community women.	1. The evaluation found that both versions of the Entre Nosotras intervention were safe, acceptable, and appropriate for participants. Challenges included slow recruitment and high attrition rates due to COVID-19, population mobility, and competing responsibilities.<br>2. Psychometric performance of psychosocial outcome measures was mixed, and intervention completion rates varied across conditions and communities<br>3. The study highlights the feasibility of combining locally designed intervention elements with evidence-based components, suggesting that the model may be applicable in different humanitarian settings.
Greene et al., 2024	To assess the feasibility, acceptance, and initial effectiveness of interpersonal counseling (IPC-	A Mixed-Methods Pilot Study.	**Setting:** Peru<br>**Population:** Venezuelan refugees and migrants	1. Study results show that Venezuelan immigrants and refugees in Peru experienced significant reductions in depression, anxiety, post-traumatic stress symptoms, and functional impairment after IPC-3.

Extraction Table

	3) among Venezuelan immigrants and refugees living in Peru.			2. Participants reported improved mental health and a more positive attitude towards life.<br>3. Qualitative data supported these findings, highlighting the impact of the intervention on mood, confidence and overall well-being.
Hajjar & Abu-Sittah, 2021	To assess the burden of COVID-19 by looking at the current living conditions, examining available services provided, and identifying the economic and health challenges of Syrian refugee families.	Qualitative study: This is a cross-sectional study of interviews conducted on on 129 Syrian refugee families	**Setting:** Lebanon<br>**Population:** Syrian refugees	1. Economic hardship, limited access to healthcare, social isolation, and increased anxiety and stress are some of the challenges reported.<br>2. The study also found that the pandemic disproportionately impacted women and children, who faced additional challenges related to caregiving responsibilities and limited access to education.
Hall et al., 2021	To explore how the COVID-19 pandemic has affected the mental health and well-being of international migrants in China. This includes examining depressive symptoms, quality	A cross-sectional study.	**Setting:** China<br>**Population:** International migrants.	1. It found that African migrants and business migrants had worse mental health compared to others.<br>2. Poor attitudes towards COVID-19 and lack of confidence were linked to higher depression and lower QoL. Positive social interactions and trust in Chinese institutions improved well-being. Alcohol misuse was influenced by COVID-19 attitudes but less by social support or trust.

Extraction Table

	of life, and alcohol misuse.			3. The results highlight the need for targeted public health interventions for migrants.
Hoffman et al., 2023	To investigate the impact of COVID-19 stressors on the mental health and well-being of refugees who are experiencing sustained displacement in Indonesia	Quantitative: Survey of 913 refugees and asylum seekers, analyzed through latent class analysis.	**Setting:** Indonesia **Population:** Refugees and asylum seekers	1. The study found that COVID-19 stressors were associated with increased symptoms of anxiety, depression, and post-traumatic stress disorder (PTSD) among refugees. 2. The study also found that refugees experienced additional stressors related to COVID-19, including increased economic insecurity and reduced access to mental health services. 3. Finally, the study highlighted the importance of social support and coping strategies for protecting refugee mental health during the pandemic.
Hossain et al., 2022	To investigate the risk factors, contemporary challenges, and psychological well-being of the Rohingya refugees	Qualitative: commentary/ perspective paper	**Setting:** Rohingya refugee camps in Bangladesh **Population:** Rohingya refugees	1. Food insecurity, inadequate shelter, limited access to healthcare, and restricted mobility are some of the numerous challenges faced by the refugees. These challenges were found to contribute to the poor psychological well-being of the refugees, including high levels of anxiety, depression, and post-traumatic stress disorder (PTSD) symptoms. 2. The study also identified risk factors for poor mental health among the Rohingya refugees, including gender, age, education, marital status, and trauma exposure.
İkiışık et al., 2022	To assess the perception of risk	A cross-sectional study.	**Setting:** Turkey	1. This study reveals that Syrian refugees in Istanbul face high unemployment, limited

Extraction Table

	of COVID-19 in the refugee populations in Turkey, in addition to evaluating anxiety levels during the pandemic.		**Population:** refugees	healthcare access, and significant anxiety during COVID-19.<br>2. Although they perceive a lower risk of infection, barriers like financial constraints and language issues hinder effective self-protection.<br>3. The findings stress the need for culturally sensitive health interventions, better access to resources, and additional financial support for refugees, with future research focusing on vaccine uptake and long-term mental health impacts.
Jamal et al., 2022	To determine the effects of the COVID-19 pandemic on the mental health of Palestine refugees in Gaza by identifying the role of social determinants.	A qualitative study conducted with interviews.	**Setting:** Rafah and Jabalia refugee camps in Gaza<br>**Population:** Palestine refugees	1. The relationship between COVID-19 and living conditions may be unidirectional, with COVID-19 causing the degradation of living conditions and vice versa, with living conditions exacerbating the COVID-19 situation by facilitating virus transmission.<br>2. The association between mental health determinants and COVID-19 may be bidirectional.<br>3. Women, children, and daily-paid employees were significantly more disadvantaged than other groups in the community in terms of experiencing violence and anxieties.
Jones at al., 2022	To explore how the COVID-19 pandemic and its associated risk mitigation strategies	A mixed-methods design.	**Setting:** Jordan<br>**Population:** internally displaced refugees and host	1. The study found that the COVID-19 pandemic and its associated risk mitigation strategies significantly exacerbated existing social inequalities among adolescents in humanitarian settings.

Extraction Table

	exacerbate existing social inequalities between internally displaced refugees and host community adolescents in Jordan.		community adolescents	2. Adolescents living in host communities and informal tented settlements were more negatively affected than those living in refugee camps.<br>3. Mitigating the impacts of the pandemic on adolescents' developmental trajectories may require multisectoral cooperation and multi-level programs that attend to priority needs of the most vulnerable adolescents, including food insecurity, gender- and age-based violence, and mental ill-health.
Khai, 2023	To examine how the military coup exacerbated the health and well-being of IDPs. The researchers investigated the primary challenged by IDPS and how those challenges impacted mental health and well-being.	Qualitative, online and telephonic interviews. Participants included 7 camp leaders and 10 IDPs.	**Setting:** Myanmar (Chin, Karen, and Kahin states)<br>**Population:** Internally Displaced Persons (IDPs)	The study found that IDPs were experiencing constant fears and anxieties due to the escalation of armed conflict. The military performs random raids on innocent civilians, which can result in sexual violence, torture, or death. The civilians also fear the frequently occurring heavy weapons attacks, which harm the mental health and well-being of the IDP population. Civilians are in a constant state of fear, with additional concern for children and women due to the history of sexual abuse perpetrated by the military. Participants are also experiencing hopelessness about returning to their homes.
Khozaei et al., 2022	To examine the role of perceived justice, freedom and the burden of COVID-19 on experienced stress and depression	A Focus group of 497 participants representing the Afghan migrant community between 15 and 80 years old	**Setting:** Kerman, Iran<br>**Population:** Afghan migrants	1. The results show that stress mediates the effect of justice, freedom and the burden of COVID-19 on depression.<br>2. In addition, positive mental health moderates the impact of stress on depression

Extraction Table

	among Afghan migrants in Iran.	participated in the study.		
Khozaei et al., 2024	The study examines the role of perceived justice, freedom, and the burden of Covid-19 on experienced stress and depression among Afghan migrants in Iran. Specifically, the study looks at how the burden of Covid-19 and the participants' perceptions of justice and freedom are associated with stress and depression, as well as how stress affects depression and how positive mental health moderates that relationship.	Survey study completed by 497 afghan participants. Questionnaire included several scales that measure different aspects of mental health (including depression and stress, positive mental health, justice and freedom, and the burden of Covid-19)	**Setting:** Kerman, Iran **Population:** Afghan migrants	Study found that the burden of Covid-19 increased migrants stress, while perceived justice and freedom had a negative effect on stress. The stress of COVID had a positive effect on depression, while perceived justice had a negative effect on depression. There was no significant effect of perceived freedom on depression. Stress mediates the effect of perceived justice, freedom and the burden of COVID-19 on depression. Positive mental health moderated the impact of stress on depression.
Kira et al., 2021	Test the direct and indirect effects of Covid-19 traumatic stress	Survey study completed by 411 Syrian refugees. Questionnaires	**Setting:** Turkey (Kilis and Hatay) **Population:**	COVID-19 fears, economic and social sub traumas have direct effects on increased deficits in working memory and inhibition (economic trauma has the largest effect size). It also has

Extraction Table

	and its three components (fear, economic, and social traumas) on working memory and inhibition.	included the Covid-19 trauma stress scale, the adult executive functioning inventory, the cumulative stressors and trauma scale, the posttraumatic stress disorder checklist for DSM-5, the General Anxiety Disorder-7, and the patient health questionnaire-9	Syrian refugees	indirect effects on it which is mediated by PTSD and depression. Additionally, Individuals that are hospitalized due to Covid-19 have higher working memory deficits that those hospitalized for other reasons.
Kira et al., 2023	To validate the Type III continuous traumatic stress model assumptions and use it to assess the effect of intersected traumas of civil war and COVID-19 stressors on internally displaced Syrian people.	A cross-sectional design, where data was collected from 891 internally displaced Syrians.	**Setting:** Idlib and Aleppo governorates close to the Syrian–Turkish borders **Population:** IDP Syrians	1. The study found that exposure to Type III traumatic stressors, such as protracted conflict and prolonged COVID-19, was associated with higher psychological distress, anxiety, and depression among internally displaced Syrians. 2. The study also found that social support and resilience played important roles in buffering the negative effects of these traumatic stressors on mental health

Extraction Table

Kizilhan and Noll-Hussong, 2020	The purpose was to compare the change in mental health before and during COVID.	Pilot study completed by 68 Yazidis before COVID and during COVID. Questionnaires included demographics, the Revised Impact of Event Scale, and the Composite International Diagnostic Scale	**Setting:** Duhok, Iraq **Population:** Refugees	Study showed a significant effect for the sub-domains of PTSD including 'reexperiencing the event, 'avoidance and numbness of feelings, 'hyperarousal,' and 'repercussions of the preceding symptoms on activities of daily living.' The prevalence of PTSD was 58% in females and 47% in males.
Kurt et al., 2021	Investigate the psychological impacts of Covid-19 related stressors, resource loss, and percieved discrimination during the pandemic as well as the buffering role of percieved social support against the determintal effects.	Online cross-sectional survey study completed by 345 Syrian Refugees. Questionnaires included the conservation of resources evaluation, everyday discrimination scale, multidimensional scale of percieved social support, patient health questionnaire-9, and the	**Setting:** Turkey **Population:** Refugees.	Authors found that high level of anxiety and depressive symptoms in sample. Participants experiencing resource loss significantly predicted higher symptoms of anxiety and depression among Syrian refugees. Participants perceived discrimination during the pandemic predicted a higher level of depressive and anxious symptoms. Higher perceived support predicted a lower level of anxiety and depressive symptoms. When perceived social support was low, resource loss predicted psychological distress. When perceived social support was high, resource loss was not a predictor of psychological distress.

Extraction Table

		generalized anxiety scale.		
Lee et al., 2022	To investigate the implementation of a community-based psychosocial support focal point response for internally displaced persons (IDPs) in Myanmar during the COVID-19 pandemic	Mixed-methods: evaluation of Community-based psychosocial support focal point response, based on program reach and utility. Mixed (qualitative and quantitative) methods were used to collect data.	**Setting:** Myanmar **Population:** internally displaced persons (IDPs)	1. The Psychosocial support (PSS) focal points were successful in distributing PSS materials to a broad set of IDP households and were recognized as points of contact for mental health counseling services.<br>2. The PSS materials provided were found to be useful and accessible for community members to understand and practice the skills<br>3. The program has been adapted to include more general stress and trauma examples and address concerns related to the coup<br>4. Adaptations to the program may need to be made to match the context of new areas, including a review of individual access to personal phones and identifying appropriate frontline workers.
Lushchak et al., 2024	Investigate the prevalence of anxiety, depression, and PTSD among Ukranians after the first year of Russian Invasion. The study included not displaced persons, internally	Online cross-sectional survey completed by 1954 Ukranians (505 IDP and 714 refugees). Survey included the Percieved Stress Scale-10, the GAD-7, and the PTSD checklist for DSM-5	**Setting:** Various countries, including Ukraine. **Population:** IDP and refugee populations.	Refugees had the highest stress levels, followed by IDPs and NDPs. Refugees and IDPs has the highest level of anxiety when compared to NDPs. Refugees has significantly higher prevalence of PTSD then IDPs, and IDPs has significantly higher prevalence of PTSD compared to NDPs.

Extraction Table

	displaced persons, and refugees.			
Mahmoodi et al., 2023	To investigate the effect of social determinants of health on the quality of life among Afghan refugees in Iran during the COVID-19 pandemic.	A cross-sectional study conducted on 300 Afghan refugees and migrants. Quantitative: Questionnaire conducted on a focus group.	**Setting:** Alborz province, Iran **Population:** refugees and migrants	1. Social determinants of health, including economic status, education level, and social support, significantly impacted the quality of life among Afghan refugees in Iran during the COVID-19 pandemic. 2. The study also found that refugees who reported higher levels of social support and education had a better quality of life outcomes, while those who reported lower economic status had worse outcomes. 3. The authors concluded that interventions to improve the social determinants of health, including access to education and economic opportunities, and to promote social support among Afghan refugees in Iran were essential for improving their quality of life during the COVID-19 pandemic
Manirambona et al., 2021	To explore the impact of the COVID-19 pandemic on the food rations provided to refugees in Rwanda	Qualitative: Questionnaire cross-sectional design, where data was collected from 891 internally displaced Syrians. Data was analyzed using hierarchical	**Setting:** Great Lakes Refugee camps in Rwanda **Population:** Refugees	Reduction of food aid by 60% in February of 2021. Replacement of food assistance with unconditional cash transfers was insufficient to meet the needs of refugees. This led to psychological distress and forced returns

Extraction Table

		multiple regression.		
Miller et al., 2022	To evaluate the caregiver support intervention for Syrian refugees	Guided focus group discussion. Participants included 122 intervention participants.	**Setting:** Two communities in North Lebanon **Population:** Syrian refugees	Participants that completed the intervention described being less angry and more patient with their children.
Mohammadsadeghi et al., 2022	To investigate experiences of Afghans early before, during, and after immigration and the pandemic's challenges to their lives in Iran.	Semi-structured Interviews with 10 Afghans who either immigrated legally or illegally.	**Setting:** Iran **Population:** Afghan immigrants and displaced persons.	Three main themes were extracted:<br>1. The tsunami of suffering was the main theme as there were multiple areas of suffering<br>2. Feelings of being lost in space while escaping Iran<br>3. Feeling of changing from a citizen to refugee and feeling discriminated and stigmatized against.
Mukumbang, et al., 2020	To discuss how COVID-19 has worsened existing vulnerabilities of asylum-seekers, refugees, and undocumented migrants	Quantitative: cross-sectional study conducted on 300 Afghan refugees and migrants with scales for socioeconomic status, World Health Organization's quality of life - BREF (WHOQOL), Depression, Anxiety and	**Setting:** South Africa **Population:** Asylum-seekers, refugees, and undocumented migrants	Social exclusion, food and housing insecurity, unemployment poorly, unequal access to health care, and regularized asylum systems lead to negative coping strategies, causing mental health issues and secondary health concerns.

Extraction Table

		Stress Scale—21 Items (DASS-21), and COVID-19 Post-Traumatic Stress Disorder Checklist (COVID-PTSD). Data was analyzed using path analysis.		
Mumin et al., 2022	To investigate the impact of COVID-19 on the livelihoods, food security and mental health of Somalia's IDPs	A cross-sectional study qualitative study.	**Setting:** Somalia **Population:** IDPs	1. COVID-19 and its prevention measures had a significant impact on the livelihoods, food security, and mental health of IDPs in Somalia 2. The participants reported experiencing reduced income and job loss due to COVID-19 prevention measures, as well as increased food insecurity and reduced access to healthcare 3. The COVID-19 pandemic had a negative impact on the mental health of IDPs, with participants reporting increased anxiety, depression, and stress 4. Interventions to mitigate the impact of COVID-19 on the livelihoods, food security, and mental health of IDPs were urgently needed, and these interventions should be integrated into broader efforts to address the root causes of displacement and improve the overall well-being of the IDP population in Somalia
Oyekale, 2022	To analyze the effect of mental health disorders and indicators of COVID-19 preventive	Quantitative: questionnaire conducted with "breadwinners" using the WHO Wellbeing Index	**Setting:** Kakuma, Kalobeyei, Dadaab and Shona refugee camps in Kenya	1. The study found that refugees in Kenya had high levels of mental health disorders, with nearly half of the participants reporting symptoms of anxiety and depression.

Extraction Table

	practices on vaccination intentions among refugees in Kenya	and the Patient Health Questionnaire. Data was analysed using Multivariable regression.	**Population:** Refugees	2. The study also found that COVID-19 prevention compliance was relatively low among the refugees, with less than half of the participants reporting consistent use of masks and social distancing.<br>3. Vaccination intentions were also relatively low, with less than half of the participants expressing a willingness to be vaccinated against COVID-19.
Ozer et al., 2022	To compare the living conditions of IDPs before and during the lockdown implemented by the authorities to contain the spread of the virus	Exploratory qualitative study: Interviews were conducted with 106 IDPs in Kongoussi (Central-Northern region)	**Setting:** Ouagadougou, Burkina Faso<br>**Population:** IDPs	1. For most of them, their living conditions, already described as difficult under 'normal' circumstances (insufficient food, insignificant financial assistance, or difficult access to health care), further deteriorated.<br>2. In addition, IDPs could not leave the camps or regions where they were located to search for better living conditions or to return home.<br>3. Lastly, the respondents believed that the COVID-19 pandemic would have a negative impact on their future.
Palit et al., 2020	Investigate the impact of Covid-19 on Rohingya refugees living in Bangladesh.	A longitudinal survey completed by 342 Rohingya refugees. Refugee Health Screener-15 was used during the first and follow up survey. COVID-19-Impact on Quality of Life	**Setting:** Kutupalong refugee camp in Cox's Bazar, Bangladesh<br>**Population:** Rohingya refugees	In comparison with the initial survey refugees were experiencing significantly higher levels of emotional distress and worst mental health during the follow up survey. The pandemic strongly impacted the QoL of the refugees and contributed significantly to the deterioration of their mental health. In general Rohingya women were affected significantly more than men

Extraction Table

		was also used in the follow up survey.		
Palit et al., 2022	To examine the impact of the current pandemic on the mental health of Rohingya refugees living in Bangladesh	In-depth one-to-one interviews with 30 research participants using WhatsApp. Data was analyzed using a thematic approach with an interpretive phenomenological analysis.	**Setting:** Rohingya refugee camps in Cox's Bazar, Bangladesh **Population:** Rohingya refugees	1. The analysis revealed a significant increase in mental distress among Rohingya refugees after the onset of the COVID-19 pandemic, impacting their QoL and contributing to the deterioration of their mental health<br>2. The situation worsened due to the added burden of lockdowns and the disease in a protracted environment with a scarcity of basic human requirements.<br>3. Rohingya women were significantly more affected than men, possibly because most of the Rohingya families are headed by women who had to endure the most stress to ensure the basic survival of the family members
Piñeiro & Ibarra, 2022	Investigate the mental health of immigrants (and refugees) that are stranded in one of four border cities in Mexico.	Semi-structured, in depth, face-to-face interviews completed by 57 Central American and Caribbean immigrants stranded in Mexico due to the pandemic.	**Setting:** Four border cities in Mexico: Tijuana, Juarez, and Matamoros **Population:** Central American and Caribbean immigrants	Identified five reoccurring themes around mental health<br>1. The pandemic's psychological impact, such as life in the border cities becoming more expensive<br>2. The uncertainty of being stranded in Mexico and the long wait.<br>3. Their fear of violence over the fear of contagion.<br>4. The perceived leniency of Mexico with the pandemic when compared to their countries of origin, and<br>5. Their beliefs about the pandemic and vaccines as many of them were

Extraction Table

				vaccinated but most were all willing to put aside their personal beliefs about the vaccine.
Sakyi & Amoako Johnson, 2022	Investigate the mental health of immigrants (and refugees) that are stranded in one of four border cities in Mexico.	Qualitative: Interviews were conducted with 106 IDPs in Kongoussi (Central-Northern region).	**Setting:** Ghana **Population:** Immigrants and refugees	High levels of anxiety, socio-demographic characteristics including marital status and age of the head of household were positively associated with having a severe psychological impact. However, when COVID-19 related factors, such as high knowledge of the disease and adherence to the preventive measures and pre-existing health conditions were taken into account, gender was insignificant, but other socio-demographic covariates were significant.
Secinti et al., 2023	Investigate the mental health of immigrants (and refugees) that are stranded in one of four border cities in Mexico.	Semi-structured, in depth, face-to-face interviews completed by 57 Central American and Caribbean immigrants stranded in Mexico due to the pandemic.	**Setting:** Turkey **Population:** Immigrants and refugees	The number of previous events had an effect on the severity of PTSD. Majority of participants experiences more secondary effect (such as socioeconomic stressors) than primary effects. Only some of the primary effects of Covid-19 were related to PTSD.
Seruwagi et al, 2022	To explore the association between psychological distress and social support among conflict refugees in urban, semi-rural and rural	Qualitative: Longitudinal study using surveys at different points in time. Various statistical analyses, including t-test,	**Setting:** Urban, semi-rural, and rural settlements in Uganda **Population:** Refugees	1. The study found that conflict refugees in Uganda's three settlement types (urban, semi-rural, and rural) experienced high levels of psychological distress.<br>2. The level of psychological distress was higher among female refugees than males and refugees who reported poor physical health.

Extraction Table

	settlements in Uganda during the COVID-19 pandemic	chi-square test, and stepwise linear regression were used.		3. Social support was found to be a protective factor against psychological distress among conflict refugees in Uganda<br>4. The level of social support was higher among refugees in rural and semi-rural settlements compared to those in urban settlements<br>5. The study suggests that interventions aimed at improving mental health among conflict refugees in Uganda should focus on addressing the gender-based differences in psychological distress, improving physical health, and increasing access to social support, especially in urban areas
Sevinc et al., 2021	Compares the Beck depression Inventory (BDI) scores of Syrian refugees and Turkish maintenance hemodialysis patients during the Covid-19 pandemic.	Cross-sectional design. BDI questionnaire was completed by 119 patients (75 Turkish and 22 Syrian). Demographic and clinical data was retrospectively collected from patient files for patients who filled out the questionnaire. BDI score of more than 14 was	**Setting:** Turkey **Population:** Syrian refugees and Turkish maintenance hemodialysis patients	Syrian refugees patients presented significantly higher BDI scores and had more depressive symptoms than Turkish patients.

Extraction Table

		used as a cut off for suspicion of depression.		
Shayo et al., 2024	Investigate the experiences of teachers and students on school closures due to covid-19.	In-depth interviews, completed by 44 individuals (29 teachers and 15 students).	**Setting:** Nyarugusu refugee camp, located in Kigoma Region in the Western part of Tanzania **Population:** Refugee	1. The pandemic was frightening to everyone and participants understood the importance of school closures. 2. Some participants did not perceive any negative impacts due to Covid-19, but there were also negative consequences reports such as an increase of fear, anxiety, and depression among teachers and students, and deterioration of students' progress in school. 3. School closures were widely perceived to have contributed to gender-based violence. 4. The majority of participants were looking forward to school re-opening although there was a high rate of student drop out.
Singh, 2021	To identify and synthesize the findings of the existing literature that highlighted the psychosocial and mental health issues of the migrants during the COVID-19 pandemic.	Qualitative: Structured, telephone interviews of 350 migrants and a final 297 migrants analyzed with the graphical analysis of univariate and bivariate analyses.	**Setting:** India **Population:** Vulnerable migrants	1. Psychosocial issues found among migrants were living conditions, basic needs, family concerns, and joblessness<br><br>2. The mental health issues among migrants during the COVID-19 pandemic included psychological distress, depressive disorders, anxiety disorders, and substance use disorders.<br>5.
Tay & Balasundaram, 2021	To examine the UNHCR's capacity in	Qualitative: commentary paper based on	**Setting:** Malaysia **Population:**	1. The Mental Health and Psychosocial Support (MHPSS) framework is built on three focuses: social consideration in

Extraction Table

	building mental health assistance for refugees and asylum seeks in Malaysia.	existing UNHCR public health responses and statistics.	Refugees and Asylum-seekers	basic services and community, strengthening community and family support, focused psychosocial support, and clinical service. A combination of support and specialist services, with functioning referral pathways in between is needed to scale MHPSS to serve refugee and asylum seekers in the pandemic.<br>2. The UNHCR launched Integrative Adapt Therapy, which included mass training and supervision of practitioners across a range of implementation platforms (clinics, schools, community and nonprofit organizations) in digital and physical spaces.<br>3. As of Nov. 18, 2020, 85% of service providers have completed the Integrative Adapt Therapy, ensuring "robust and rigorous training, quality assurance, and monitoring of performance, clinical, and implementation outcomes" that are rapidly deployed.
Ünver & Perdahlı Fiş, 2022	To examine the difference of admissions to a refugee child outpatient mental health unit before and after the Covid-19 pandemic.	Retrospective observational study on the Refugee Child Mental Health Unit	**Setting:** Istanbul, Turkey<br>**Population:** Refugee children	The difference of admissions for autism spectrum disorder, speech impairment, and enuresis was not significantly different when comparing pre-pandemic and the pandemic. External destructive behavioral disorders such as ADHD, oppositional defiant disorder, and conduct disorder significantly increased during the pandemic. internal disorders such as depressive disorders and anxiety disorders decreased significantly during the pandemic.

Extraction Table

Vasic et al., 2023	To explore the prevalence of alcohol and substance abuse.	Quantitative analyses of questionnaires	**Setting:** Serbia **Population:** Young refugees	1. Alcohol and substance use was associated with significant indicators such as older age (14-18 yrs), male gender, lower education, unaccompanied children and exposure to emotional abuse. 2. More than half of the same displayed symptoms of PTSD.
Yalcin et al., 2021	To investigate the levels and correlates of depression, anxiety, somatization and post-traumatic stress disorder symptoms of refugees and native patients with End-Stage Renal Diseases Receiving Hemodialysis.	Descriptive, cross-sectional study completed by 27 refugees and 31 native residents in Istanbul. Survey included sociodemographic and clinical questionnaire, Patient Health Questionnaire-Somatic, Anxiety, and Depressive Symptoms, and the Posttraumatic Stress Disorder Checklist for DSM-5	**Setting:** Istanbul, Turkey **Population:** Refugees and native patients	Refugee patients had significantly higher levels of anxiety and stress in comparison to the native group, however, depression and somatic anxiety did not differ among groups. In terms of psychiatric comorbidity, anxiety and PTSD were more frequent among refugee patients and depression was more frequent among native patients.
Zeid et al., 2023	The purpose was to determine the predictors of poor mental health among Syrian	Cross-sectional data from a five-wave study, where data was collected via	**Setting:** Lebanon **Population:** Older Syrian Refugees	Out of the 19 nineteen predictors from the original model, nine predictors of poor mental health remained in the final model (younger age, food insecurity, water insecurity, lack of legal status documentation, irregular employment,

Extraction Table

	Refugees aged 50 years or older. 3,229 refugees participated during Wave 1 of data collection.	telephone. Participants consisted of older Syrian refugees (50 years and older). Analysis was completed through backwards stepwise logistic regression (using wave 1 data). Nineteen posssible predictors were included in the full model. Analys was also done of the difference of mental health status accross the three waves.		higher intensity of bodily pain, having debt, and having chronic illnesses). Additionally, at wave 1, 76.7% of participants had poor mental health which significanlty increased at waves three and five.
