# Supplementary material for: Psychosocial well-being and mental health of low- and middle-income countries’ internally displaced persons and refugees during COVID-19: a systematic literature review
Source: Glob Ment Health (Camb). 2024 Dec 10;11:e122. doi: 10.1017/gmh.2024.110 (PMC11704375; doi:10.1017/gmh.2024.110)
Supplement: Amodu et al. supplementary material 2 — Amodu et al. supplementary material [file S2054425124001109sup002.pdf]

Literature Search History

Expansion to Broader LMIC Populations

The literature review searches were conducted on January 16, 2023 in five databases: MEDLINE (1946-present via Ovid) (n=413), EMBASE (n=587), CINAHL (n=186), PubMed (n=906), and PsychINFO (n=174) with the use of the following search string combinations. The searches followed the basic format of searching within the contexts of the population of study, with relation to COVID-19 and the period of time surrounding the pandemic, as well as search strings related to mental health, with the MEDBASE format provided as an example in the main text. On July 10, 2024, these searches were repeated to ensure that the manuscript was relevant in publication, using the same search strings except for PsychINFO. All database search strings are seen below.

Commented [KTP1]: This Supplementary material details the updated PRISMA diagram, methodology, and any changes in search strings.

MEDLINE

(with all search strings combined with Boolean term "AND")

- exp "Emigrants and Immigrants"/ or Refugees/ or (immigrant* or immigration or emigrant* or emigration or refugee* or "asylum seeker*" or asylee* or "displaced person*" or "displaced people" or "incomer*" or "in comer*" or "new comer*" or newcomer* or migrant* or resettler*).mp.
- (((exp Coronavirus/ or exp Coronavirus Infections/ or (coronavirus* or corona virus* or OC43 or NL63 or 229E or HKU1 or HCoV* or ncov* or covid* or sars-cov* or sarscov* or Sars-coronavirus* or Severe Acute Respiratory Syndrome Coronavirus*).mp.) and (201906* or 201907* or 201908* or 201909* or 20191* or 2020* or 2021* or 2022* 2023* or 2024* or 2025* or 2026* or 2027* or 2028* or 2029* or 2030*).dt,ez,da.) not (SARS or SARS-CoV or MERS or MERS-CoV or Middle East respiratory syndrome or camel* or dromedar* or equine or coronary or coronal or covidence* or covidien or influenza virus or HIV or bovine or calves or TGEV or feline or porcine or BCoV or PED or PEDV or PDCoV or FIPV or FCoV or SADS-CoV or canine or CCov or zoonotic or avian influenza or H1N1 or H5N1 or H5N6 or IBV or murine corona*).mp.) OR ((Covid-19/ or covid or covid19 or 2019-ncov or ncov19 or ncov-19 or 2019-novel CoV or sars-cov2 or sars-cov-2 or sarscov2 or sarscov-2 or Sars-coronavirus2 or Sars-coronavirus-2 or SARS-like coronavirus* or coronavirus-19 or Deltacron or Omnicron or ((novel or new or nouveau) adj2 (CoV or nCoV or covid or coronavirus* or corona virus or Pandemi*2)) or ((subvariant* or variant*) adj2 (India* or "South Africa*" or UK or English or Brazil* or alpha or beta or delta or gamma or kappa or lambda or mu or "AY.X" or "BA.1" or "BA.2" or "BA.3" or "BA.4" or "BA.5" or "P.1" or "C.37")) or ("B.1.1.7" or "B.1.351" or "B.1.617.1" or "B.1.617.2" or "B.1.1.529*" or "B.1.61.7*" or "21L/BA.2" or "21K/BA.1").mp.))

- ("mental* health*" or "Psychosocial wellbeing" or depress* or sadness or stress or anxiety or anxious* or PTSD or "mental* ill*" or "mental* disorder*").mp.

EMBASE

(with all search strings combined with Boolean term "AND")

- migrant/ or emigrant/ or forced migrant/ or immigrant/ or migrant worker/ or internally displaced person/ or refugee/ or asylum seeker/ or (immigrant* or immigration or emigrant* or emigration or refugee* or "asylum seeker*" or asylee* or "displaced person*" or "displaced people" or "incomer*" or "in comer*" or "new comer*" or newcomer* or migrant* or resettler*).mp.
- ((exp Coronavirus/ or exp Coronavirus Infections/ or (coronavirus* or corona virus* or OC43 or NL63 or 229E or HKU1 or HCoV* or ncov* or covid* or sars-cov* or sarscov* or Sars-coronavirus* or Severe Acute Respiratory Syndrome Coronavirus* or D614G).mp.) not (SARS or SARS-CoV or MERS or MERS-CoV or Middle East respiratory syndrome or camel* or dromedar* or equine or coronary or coronal or covidence* or covidien or influenza virus or HIV or bovine or calves or TGEV or feline or porcine or BCoV or PED or PEDV or PDCoV or FIPV or FCoV or SADS-CoV or canine or CCov or zoonotic or avian influenza or H1N1 or H5N1 or H5N6 or IBV or murine corona*).mp.) or coronavirus disease 2019/ or ((exp pneumonia/ or (pneumonia or covid* or coronavirus* or corona virus* or ncov* or 2019-ncov or sars*).mp.) and Wuhan.mp.) or ("coronavirus disease 2019" or 2019-ncov or ncov19 or ncov-19 or 2019-novel CoV or severe acute respiratory syndrome coronavirus 2 or sars-cov2 or sars-cov-2 or sarscov2 or sarscov-2 or Sars-coronavirus2 or Sars-coronavirus-2 or SARS-like coronavirus* or coronavirus-19 or covid19 or covid-19 or "covid 2019" or "B.1.1.7" or "B.1.351" or "B.1.617.1" or "B.1.617.2" or omicron or Deltacron).mp. or ((subvariant* or variant*) adj2 (India* or "South Africa*" or UK or English or Brazil* or alpha or beta or delta or gamma or kappa or lambda or mu or "AY.X" or "BA.1" or "BA.2" or "BA.3" or "BA.4" or "BA.5" or "P.1" or "C.37")).mp. or ("B.1.1.7" or "B.1.351" or "B.1.617.1" or "B.1.617.2" or "B.1.1.529*" or "B.1.61.7*" or "21L/BA.2" or "21K/BA.1").mp. or ((novel or new or nouveau) adj2 (CoV or nCoV or coronavirus* or corona virus)).mp.
- ("mental health*" or "Psychosocial wellbeing" or depression or sadness or stress or anxiety or "mental illness" or "mental disorders").mp.

CINAHL

(with all search strings combined with Boolean term "AND")

- (MH "Refugees+") OR (MH "Immigrants") OR (MH "Undocumented Immigrants") or (MH "Emigration and Immigration") OR (MH "Relocation") OR (MH "Transients and Migrants") or (immigrant* or immigration or emigrant* or emigration or refugee* or "asylum seeker*" or asylee* or "displaced person*" or "displaced people" or "incomer*" or "in comer*" or "new comer*" or newcomer* or migrant* or resettler*)

- (((MH "Coronavirus+") OR (MH "Coronavirus Infections+") or (coronavirus* or corona virus* or OC43 or NL63 or 229E or HKU1 or HCoV* or ncov* or covid* or sars-cov* or sarscov* or Sars-coronavirus* or Severe Acute Respiratory Syndrome Coronavirus*)) NOT ((SARS or SARS-CoV or MERS or MERS-CoV or Middle East respiratory syndrome or camel* or dromedar* or equine or coronary or coronal or covidence* or covidien or influenza virus or HIV or bovine or calves or TGEV or feline or porcine or BCoV or PED or PEDV or PDCoV or FIPV or FCoV or SADS-CoV or canine or CCov or zoonotic or avian influenza or H1N1 or H5N1 or H5N6 or IBV or murine corona*)) or (MH "COVID-19") OR (MH "COVID-19 Pandemic") OR (MH "SARS-CoV-2") or(covid or 2019-ncov or ncov19 or ncov-19 or 2019-novel CoV or sars-cov2 or sars-cov-2 or sarscov2 or sarscov-2 or Sars-coronavirus2 or Sars-coronavirus-2 or SARS-like coronavirus* or coronavirus-19 or ((novel or new or nouveau) N2 (CoV or nCoV or coronavirus* or "corona virus" or Pandemi*)) or ((subvariant* or variant*) adj2 (India* or "South Africa*" or UK or English or Brazil* or alpha or beta or delta or gamma or kappa or lambda or mu or "AY.X" or "BA.1" or "BA.2" or "BA.3" or "BA.4" or "BA.5" or "P.1" or "C.37")) or ("B.1.1.7" or "B.1.351" or "B.1.617.1" or "B.1.617.2" or "B.1.1.529*" or "B.1.61.7*" or "21L/BA.2" or "21K/BA.1") or Deltacron or Omnicron) and EM 20190601-20301231
- ("mental health*" OR "Psychosocial wellbeing" OR depression OR sadness OR stress OR anxiety OR "mental illness" OR "mental disorders")

PUBMED

(with all search strings combined with Boolean term "AND")

- Emigrants and Immigrants"/ or Refugees/ or (immigrant* or immigration or emigrant* or emigration or refugee* or "asylum seeker*" or asylee* or "displaced person*" or "displaced people" or "incomer*" or "in comer*" or "new comer*" or newcomer* or migrant* or resettler
- 2019-nCoV OR 2019nCoV OR COVID-19 OR SARS-CoV-2 OR ((wuhan AND coronavirus) AND 2019/12[PDAT]:2030[PDAT])
- ("mental health*" OR "Psychosocial wellbeing" OR depression OR sadness OR stress OR anxiety OR "mental illness" OR "mental disorders")

PSYCHINFO

(with all search strings combined with Boolean term "AND")

- Emigrants and Immigrants"/ or Refugees/ or (immigrant* or immigration or emigrant* or emigration or refugee* or "asylum seeker*" or asylee* or "displaced person*" or "displaced people" or "incomer*" or "in comer*" or "new comer*" or newcomer* or migrant* or resettler

- ((exp Coronavirus/ or (coronavirus* or corona virus* or OC43 or NL63 or 229E or HKU1 or HCoV* or ncov* or covid* or sars-cov* or sarscov* or Sars-coronavirus* or Severe Acute Respiratory Syndrome Coronavirus* or D614G).mp.) not (SARS or SARS-CoV or MERS or MERS-CoV or Middle East respiratory syndrome or camel* or dromedar* or equine or coronary or coronal or covidence* or covidien or influenza virus or HIV or bovine or calves or TGEV or feline or porcine or BCoV or PED or PEDV or PDCoV or FIPV or FCoV or SADS-CoV or canine or CCov or zoonotic or avian influenza or H1N1 or H5N1 or H5N6 or IBV or murine corona*).mp.) or Covid-19/ or (((pneumonia or covid* or coronavirus* or corona virus* or ncov* or 2019-ncov or sars*).mp. or exp pneumonia/) and Wuhan.mp.) or ("coronavirus disease 2019" or 2019-ncov or ncov19 or ncov-19 or 2019-novel CoV or severe acute respiratory syndrome coronavirus 2 or sars-cov2 or sars-cov-2 or sarscov2 or sarscov-2 or Sars-coronavirus2 or Sars-coronavirus-2 or SARS-like coronavirus* or coronavirus-19 or covid19 or covid-19 or covid 2019 or "B.1.1.7" or "B.1.351" or "B.1.617.1" or "B.1.617.2" or ((subvariant* or variant*) adj2 (India* or "South Africa*" or UK or English or Brazil* or alpha or beta or delta or gamma or kappa or lambda or mu or "AY.X" or "BA.1" or "BA.2" or "BA.3" or "P.1" or "C.37")) or ("B.1.1.7" or "B.1.351" or "B.1.617.1" or "B.1.617.2" or "B.1.1.529*" or "B.1.61.7*" or "21L/BA.2" or "21K/BA.1" or "BA.4" or "BA.5") or Deltacron or Omnicron) or ((novel or new or nouveau) adj2 (CoV or nCoV or coronavirus* or corona virus)).mp.
- ("mental health*" OR "Psychosocial wellbeing" OR depression OR sadness OR stress OR anxiety OR "mental illness" OR "mental disorders")

The following is an updated search of the literature within the PsychINFO database using updated search strings:

- exp immigration/ or refugees/ or (immigrant* or immigration or emigrant* or emigration or refugee* or "asylum seeker*" or asylee* or "displaced person*" or "displaced people" or "incomer*" or "in comer*" or "new comer*" or newcomer* or migrant* or resettler).mp.
- ((exp Coronavirus/ or (coronavirus* or corona virus* or OC43 or NL63 or 229E or HKU1 or HCoV* or ncov* or covid* or sars-cov* or sarscov* or Sars-coronavirus* or Severe Acute Respiratory Syndrome Coronavirus* or D614G).mp.) not (SARS or SARS-CoV or MERS or MERS-CoV or Middle East respiratory syndrome or camel* or dromedar* or equine or coronary or coronal or covidence* or covidien or influenza virus or HIV or bovine or calves or TGEV or feline or porcine or BCoV or PED or PEDV or PDCoV or FIPV or FCoV or SADS-CoV or canine or CCov or zoonotic or avian influenza or H1N1 or H5N1 or H5N6 or IBV or murine corona*).mp.) or Covid-19/ or (((pneumonia or covid* or coronavirus* or corona virus* or ncov* or 2019-ncov or sars*).mp. or exp pneumonia/) and Wuhan.mp.) or ("coronavirus disease 2019" or 2019-ncov or ncov19 or ncov-19 or 2019-novel CoV or severe acute respiratory syndrome coronavirus 2 or sars-cov2 or sars-cov-2 or sarscov2 or sarscov-2 or Sars-coronavirus2 or Sars-coronavirus-2 or SARS-like coronavirus* or coronavirus-19 or covid19 or covid-19

or covid 2019 or "B.1.1.7" or "B.1.351" or "B.1.617.1" or "B.1.617.2" or ((subvariant* or variant*) adj2 (India* or "South Africa*" or UK or English or Brazil* or alpha or beta or delta or gamma or kappa or lambda or mu or "AY.X" or "BA.1" or "BA.2*" or "BA.3" or "BA.4" or "BA.5" or "P.1" or "C.37" or "EG.5*" or "EG.5.1*" or "EG.5.1.1*" or "HK.3*" or "HV.1*" or "JG.3*" or "JN.1*")) or ("B.1.1.7" or "B.1.351" or "B.1.617.1" or "B.1.617.2" or "B.1.1.529*" or "B.1.61.7*" or "21L/BA.2" or "21K/BA.1" or "XBB.1.5 " or "XBB.1.9" or "XBB.1.16" or "XBB.2.3") or Deltacron or Omnicron).mp. or ((novel or new or nouveau) adj2 (CoV or nCoV or coronavirus* or corona virus)).mp.

- ("mental health*" or "Psychosocial wellbeing" or depression or sadness or stress or anxiety or "mental illness" or "mental disorders").mp.

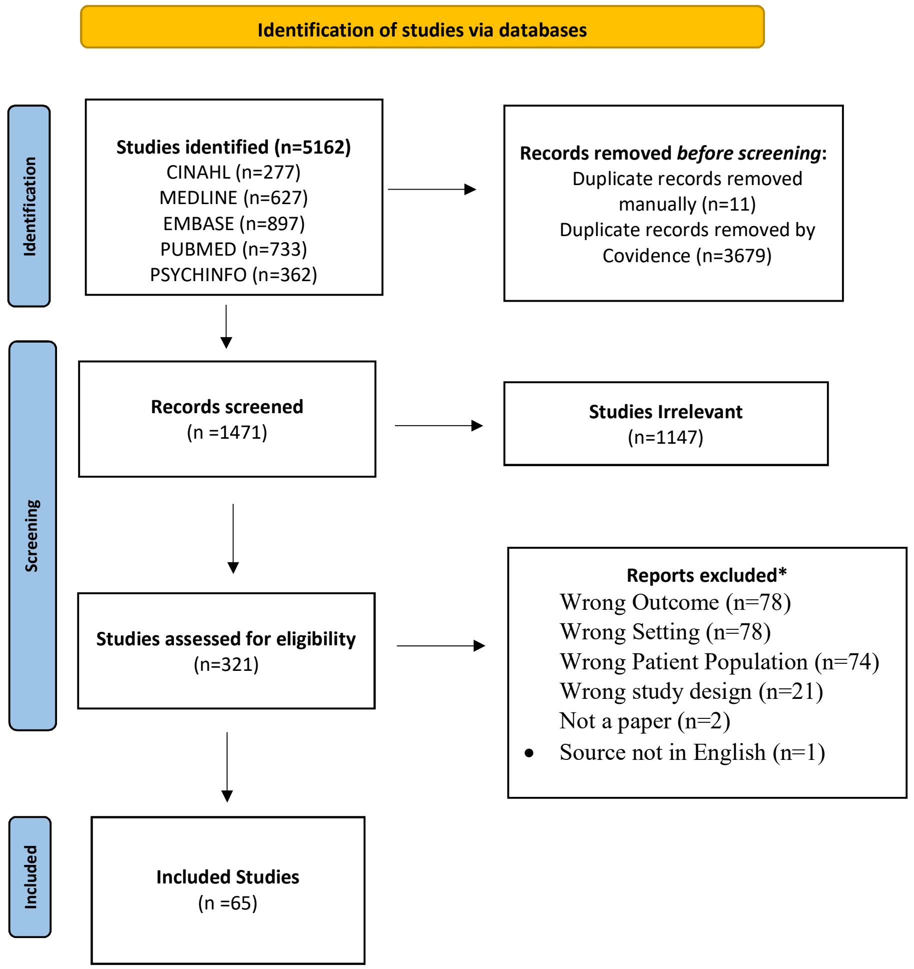

Wrong Outcome: Studies were excluded if their primary or secondary outcomes did not align with our research objectives, which focused on psychosocial and mental health issues. For example, if a study only reported physical health outcomes or biochemical markers, it was excluded.

Wrong Setting: Studies conducted in settings outside of LMICs were excluded, as our focus was on refugees, asylum seekers, and displaced persons in LMIC settings since the onset of COVID-19.

Wrong Patient Population: We excluded studies if their patient population did not match our inclusion criteria. This included studies that focused on non-refugee populations, individuals from high-income countries, or populations with a specific focus on age, religion, or race (with the exception of country of origin).

Wrong Design: Although we did not restrict the methodology, we excluded studies that did not contribute to a comprehensive, critical, and objective analysis of the current knowledge on the topic. For instance, case reports or studies lacking rigorous data analysis were excluded when more robust evidence was available.
